# Reaching young women who sell sex: Methods and results of social mapping to describe and identify young women for DREAMS impact evaluation in Zimbabwe

**DOI:** 10.1371/journal.pone.0194301

**Published:** 2018-03-15

**Authors:** Tarisai Chiyaka, Phillis Mushati, Bernadette Hensen, Sungai Chabata, James R. Hargreaves, Sian Floyd, Isolde J. Birdthistle, Frances M. Cowan, Joanna R. Busza

**Affiliations:** 1 Centre for Sexual Health and HIV/AIDS Research (CeSHHAR), Harare, Zimbabwe; 2 London School of Hygiene and Tropical Medicine, London, United Kingdom; 3 Liverpool School of Tropical Medicine, Liverpool, United Kingdom; UNAIDS, UNITED STATES

## Abstract

Young women (aged 15–24) who exchange sex for money or other support are among the highest risk groups for HIV acquisition, particularly in high prevalence settings. To prepare for introduction and evaluation of the DREAMS programme in Zimbabwe, which provides biomedical and social interventions to reduce adolescent girls’ and young women’s HIV vulnerability, we conducted a rapid needs assessment in 6 towns using a “social mapping” approach. In each site, we talked to adult sex workers and other key informants to identify locations where young women sell sex, followed by direct observation, group discussions and interviews. We collected data on socio-demographic characteristics of young women who sell sex, the structure and organisation of their sexual exchanges, interactions with each other and adult sex workers, and engagement with health services. Over a two-week period, we developed a “social map” for each study site, identifying similarities and differences across contexts and their implications for programming and research. Similarities include the concentration of younger women in street-based venues in town centres, their conflict with older sex workers due to competition for clients and acceptance of lower payments, and reluctance to attend existing services. Key differences were found in the 4 university towns included in our sample, where female students participate in diverse forms of sexual exchange but do not identify themselves as selling sex. In smaller towns where illegal gold panning or trucking routes were found, young women migrated in from surrounding rural areas specifically to sell sex. Young women who sell sex are different from each other, and do not work with or attend the same services as adult sex workers. Our findings are being used to inform appropriate intervention activities targeting these vulnerable young women, and to identify effective strategies for recruiting them into the DREAMS process and impact evaluations.

## Introduction

Adolescent girls and young women (AGYW) aged 15–24 are at disproportionately high risk of acquiring HIV in sub-Saharan Africa; they accounted for 25% of new adult HIV infections in 2015 [1]. Young women who sell sex (YWSS) face particularly high risks due to high numbers of sexual partners, inability to negotiate condom use, and limited access to health services[2, 3]. Survey and programme data collected from female sex workers across Zimbabwe since 2009 suggest that that YWSS exist in sizeable numbers, and around 20% of female sex workers (FSW) report initiating sex work before age 20 [4–6]. Sex work is criminalised in Zimbabwe, although increasingly ignored by the police [7].

To reduce HIV among AGYW, the DREAMS initiative (Determined Resilient Empowered AIDS-free Mentored and Safe), targets biological, behavioural, and socio- economic determinants of risk through a comprehensive package of HIV prevention interventions in 10 African countries. In Zimbabwe, DREAMS is being implemented across six districts, delivered through local implementing partners that provide access to condoms, contraception, and HIV testing, and offer educational opportunities, vocational training, and work experience to address AGYW’s socio-economic vulnerabilities. Furthermore, DREAMS is making oral pre-exposure prophylaxis (PrEP) available to YWSS aged 18–24 in four of its programme districts in Zimbabwe to ameliorate their heightened risk.

To assess the effectiveness of the DREAMS initiative over three years, two intervention (Bulawayo and Mutare) and four comparison (Chinhoyi, Karoi, Kwekwe and Zvishavane) sites were selected for inclusion in impact and process evaluation studies [8]. Prior to baseline data collection and introduction of the DREAMS package, we conducted a rapid needs assessment in each of the six sites to deepen understanding of the local context. For both intervention and comparison sites, knowledge of where and how YWSS sell sex, their relationships with each other and with adult FSW, whether they are mobile between the sites or remain “clustered”, and how they themselves perceive their involvement in exchanging sex for money or other material goods was a prerequisite for the appropriate intervention and evaluation design.

In this paper, we describe the “social mapping” approach used during the rapid needs assessment that combined identification of geographical locations where YWSS sell sex with additional data on the social context in which sex is exchanged. The aim of the study was to “paint a picture” of the YWSS physical and social environment in each study site to prepare for the DREAMS intervention and evaluation. Specific objectives of social mapping were to: 1) understand the organization and structure of commercial sex provided by YWSS and effects on their vulnerability; 2) explore YWSS social networks and levels of support; and 3) determine whether YWSS would be willing to participate in research activities including baseline and follow-up surveys and, in the intervention sites, their interest in accessing activities planned as part of DREAMS. By describing how this structured, rapid appraisal identified similarities and differences between sites, we highlight key lessons that could be useful for other programmes attempting to reach this marginalised and often hidden key population.

## Methods

We conducted social mapping in all sites selected for research to evaluate delivery and effectiveness of the DREAMS programme, including two DREAMS implementation sites (the cities of Bulawayo and Mutare) and four smaller towns designated as comparison sites (Chinhoyi, Karoi, Kwekwe and Zvishavane). All six sites provide targeted clinical services, peer education, and community mobilisation meetings for FSW as part of the *Sisters with a Voice* national sex worker HIV prevention and treatment programme. The *Sisters* programme is delivered by the Centre for Sexual Health, HIV and AIDS Research (CeSHHAR), which is also one of the organisations responsible for recruiting and referring YWSS into DREAMS activities, where available. In collaboration with the London School of Hygiene and Tropical Medicine, CeSHHAR will conduct the process and impact evaluation of DREAMS Zimbabwe.

We collected social mapping data across each site, comprising the city or town’s central business district (CBD) and all surrounding residential and industrial areas mentioned as sites where YWSS sell sex. We defined YWSS as women aged between 18–24 who exchanged sex for money or material goods, including those who self-identify as FSW as well as those who do not consider themselves as sex workers but report sex with men that would not occur in the absence of receiving financial or other resources. DREAMS targets the 10–24 age group, but only YWSS aged 18 and above are eligible for PrEP. The Zimbabwe Medical Research Council allows young people aged eighteen and above to provide consent to take part in research. Ethical approval for the DREAMS evaluation research was obtained from the Medical Research Council of Zimbabwe (MRCZ/2085) and the London School of Hygiene and Tropical Medicine (11835).

The mapping process started with informal discussions with locally deployed peer educations from the *Sisters* programme to gain an initial understanding of the geographical spread of YWSS, particularly where YWSS could be found at different times. Once a basic geographical picture was built up, a team of 3–4 female fieldworkers visited each site mentioned. Fieldworkers were CeSHHAR research staff with extensive experience of both quantitative and qualitative data collection with sex workers.

Venues included bars, hotels, guesthouses and lodges, nightclubs, markets, streets, bus/truck stops and taxi ranks. The fieldwork team visited each suggested location on the three “busiest days” of the week, Thursday, Friday and Saturday over two consecutive weekends. During site visits, we used three rapid assessment methods: direct observation, group discussions, and informal interviews. Table 1 summarises information collected by each method.

**Table 1:** Data collection methods.

Method	Sample	Type of Data Collected
Direct Observation	Visits to bars and entertainment areas. 5 nights and 5 days in Bulawayo and Mutare; 4 nights and 4 days in Karoi, Kwekwe, Zvishavane and Chinhoyi.	Numbers of sex workers and age distribution. Types and numbers of male clients. Hours of busiest activity.
Group Discussions	7 groups discussions with a total of 80 YWSS (range 6–25 participants)	Where YWSS work in the local community Differences between YWSS and adult FSW Social interactions between YWSS YWSS health challenges Use of health services Willingness to engage in research Willingness to participate in DREAMS
Informal Interviews (Uncounted and unrecorded)	YWSS Adult FSW Taxi drivers Bar tenders Managers of hotels University students Street vendors Health providers/ NGO staff	How YWSS start selling sex Organisation and prices of sex work Differences between YWSS and FSW Relationship to older FSW How clients are solicited Where sex takes place Fees charged Other locations where YWSS can be found

Fieldworkers were trained to naturally engage with the “scene” at each site, and to approach people in a friendly manner, providing their affiliation with the *Sisters* programme, which was familiar to many FSW and thus helped reduce anxiety. Fieldworkers explained the purpose of the social mapping and briefly chatted to all individuals encountered during the observation. Conversations were conducted in the local language (Shone or Ndebele) and notes were taken throughout the observation. No identifying information was recorded. Fieldworkers were encouraged to conduct “natural” conversations without topic guides. In addition to YWSS, we talked to key informants from the community including older FSW, college and university students, owners or employees of bars and other large entertainment venues (including nightclubs), hotels and guesthouses, street vendors (particularly near bars), taxi drivers, and health service staff/NGO workers who provide services to sex workers. All informants were asked to suggest additional locations we could visit to observe YWSS.

During these observations we identified YWSS to take part in group discussions and individual interviews. These discussions included both young women who openly sell sex and those who do not self-identify as sex workers but explicitly exchange sex for money or material goods. One discussion was held in each of Mutare (n = 17), Zvishavane (n = 7), Kwekwe (n = 6), Chinhoyi (n = 10) and Karoi (n = 7). Two groups were organised in Bulawayo (n = 25+15). Individual interviews were conducted with young women identified by peer educators and other community informants, and were selected for interview because of their openness during group discussions, irrespective of whether they had previously attended the Sisters clinic. They were recruited from different venues, reflecting different means for locating clients or boyfriends. All participants for group discussions and interviews provided verbal consent. As before, only written notes were taken for analysis and no identifying information recorded.

In keeping with the rapid nature of the assessment, we conducted basic content analysis of notes from observations, group discussions and interviews over several days following social mapping. For each site, we developed a detailed matrix depicting characteristics of the different venues and YWSS working there, and summarizing the number and type of respondents from whom data were collected, to ensure reliability across sites. A de-identified example is provided as Table 2, to avoid inadvertent disclosure of specific venues where YWSS sell sex which could then be targeted by unwanted publicity or police action. These tables were used to help plan for appropriate ways of reaching YWSS in intervention sites for referral into DREAMS, and to check for basic comparability across intervention and comparison sites.

**Table 2:** Sample YWSS venue observation matrix.

	Sex Work venues/ Typologies			YWSS numbers
Geographic Location	Bars/Nightclubs	Markets/Streets	Truck stops/ Highway	
**Central Business District (CBD)**	A bar Local Nightclubs (20 YWSS)–Very busy Saturday from 10pm. One club where most YWSS go. Sports club (30 young girls)—a popular and upmarket night club.	Many YWSS (18 to mid-20s) observed standing in pairs by upmarket retail stores in the CBD. Sex workers of all ages soliciting in the street.	No haulage truck stops in CBD. 5km from CBD there is 1 truck stop, BBQ grills, bottle store and butchery.	Over 50 young women selling sex observed
**Farm**	Area has 1 bar and large supermarket/ butchery. Popular with sex workers of all ages. Quiet during the week and conducive for those wanting anonymity. Short time sex is common, cost $1-$5. Short time done in the bush surrounding the venue.		No truck stop on the farm	2 sex workers 1 key informant—bar tender said when the place is busy almost 50 young women would be there
**High Density Suburb**	4 bars and 1 nightclub at a shopping centre. Some YWSS not obviously working as such, standing outside bars. Most were young. Clients here are varied, taxi drivers, vendors, policeman, soldiers, young men. YWSS move between bars.	Street SW mainly young. Stand in dark areas away from view. Short time sex in street costs $1-$3. Night costs $8—and $10. Clients are passers-by and men already known to the street based young women selling sex.	High density transport hub—taxis, minbuses, long distance buses. Market vendors and young girls pretending to be boarding buses but actually soliciting clients	About 30 young women selling sex Informal interviews held in the car
**Shopping Centre in town’s outskirts**	**Recreation parks and night clubs** 8 kilometres from the CBD, surrounded by rural area, 5 bars and 2 nightclubs serve the rural community and gold panners. The nightclubs are popular with young sex workers and open all night.		Trucks also park at the outskirt entertainment areas and attract YWSS.	About 5 informal key informant interviews

## Results

YWSS sell sex at diverse locations and events, some of which are at the boundary of sex work and more informal transactions/ exchanges. We found a continuum in how YWSS perceived their involvement in sex work, from those who identified as sex workers to those who did not consider their sexual exchanges to be income-generating activities so much as social events with financial and material benefits. We present our findings according to whether they were common to all study sites or specific to one or more, as this has implications for comparability across sites and over time during the DREAMS evaluation study. As we move forward toward evaluating DREAMS uptake and effects in each site, and compare these to non-DREAMS locations, we will need to consider how programme components interact with the local YWSS context.

**Fig 1:**
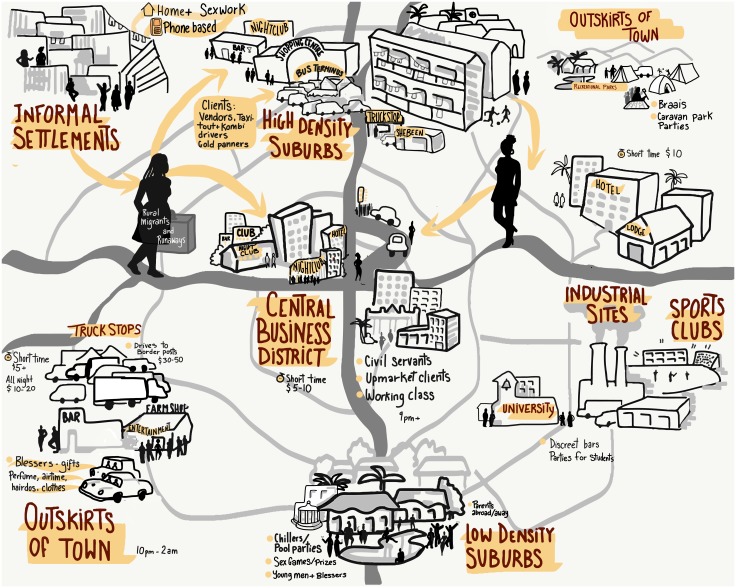
An illustration showing the geographic and sociodemographic data compiled at each site during social mapping.

We highlight findings on geographical location, YWSS characteristics, the organisation of sex work, social relationships, and uptake of and interest in services as dimensions of our “social maps”. Fig 1 provides a visual depiction of the kind of geographical and socio-demographic data compiled for each location.

### Similarities across the six sites

Across sites, there were common geographical locations where YWSS congregate. Sex work is generally concentrated in city/town centres, referred to as Central Business Districts (CBD). YWSS said they came to town from surrounding residential suburbs so that they would be unfamiliar to potential clients, and thus would be considered new and exciting. YWSS solicit clients sex in bars, nightclubs, streets, market places and leisure places (i.e. shopping centres, public parks with barbecue facilities, and entertainment zones where restaurants and bars are clustered). They have sex with clients in short-term lodges, bushes, their own homes, and in cars. YWSS’ clients are varied. Respondents mentioned taxi drivers, illegal gold panners (*makorokoza)*, civil servants, seasonal farmers, truck drivers, vendors, policeman, soldiers, and young men who are there to experiment and have fun.

CBDs proved more popular with YWSS than sex work venues in the high-density suburbs (townships), which is where most YWSS lived. This was partly so YWSS could avoid recognition by community members, but also because prices charged in city/town centres for “short time”, sex which takes under 10 minutes, were higher than out of town (US$5–10 instead of US$2–5). During group discussions, YWSS reported aiming for approximately 5 clients for “short-time” each evening, followed by looking for someone around midnight with whom to spend the rest of the night, earning $15-$20 or US$8 if ‘overnight’ started after 1 a.m.

YWSS described feeling uncomfortable inside bars with older FSW, preferring to remain outside and attract clients before they enter the bar. Most YWSS felt older bar-based sex workers looked down on and bullied them, considering them to be lower class “street” workers, and therefore rural, plain, dirty, and inexperienced. Other venues where YWSS provide sex include behind trees and in car parks. This was particularly the case when YWSS charged exceptionally low fees that were insufficient to cover the cost of accommodation. YWSS described disputes with adult FSW, particularly where older and younger sex workers directly compete and YWSS charge less. Key informant FSW over 25 years confirmed this, blaming younger women for being rowdy and badly behaved. Competition between YWSS also occurred over clients.

Outside the CBD, YWSS in all sites mentioned working in high-density suburbs and out of town venues. In the high-density areas, YWSS work the streets, lining up outside bars and around the market place. These areas are busiest on weekends and at the end of the month when clients have been paid their monthly salary. These areas host YWSS who self-identify as sex workers, and thus are less concerned about being recognised. We observed them walking up and down between bars, dressed in mini-skirts and shorts. They described charging US$ 2–5 for “short time” while a full night with a client cost $10. Venues for sex included YWSS’ homes (if they did not live with parents) and lodges. Sex also takes place on the street, popularly referred to as *mumahedges* (in the hedges) and in dark allies against the wall referred to as *pamatombo* (stones). Open spaces close to the shopping centres offer dark hidden spots where street-based sex work is popular. It emerged that in some high density areas YWSS were charging as little as $1 for sex or accepted fast food, undercutting the prices charged by older sex workers and creating the same kinds of tensions observed in the CBD.

On the outskirts of towns, public leisure areas frequented by families for barbeques and picnics by day become drinking venues at night. These are busiest when entertainment is provided, such as a live band or soccer match screening, and usually operate between 10 p.m. and dawn on Friday and Saturday nights. Older men, known as “blessers,” take young women to drink beer and dance. Young men also bring their girlfriends. These venues attract more informal and transactional sex, and many YWSS go there for their own enjoyment.

Overall, YWSS reported using their earnings to take care of their children/siblings, buy food, for beauty treatments (manicure, pedicure, hairdo), rent, school fees, and buying clothes, particularly “skimpy” outfits that their parents refuse to buy. Many YWSS live with their parents, thus aim to keep involvement in sex work secret. Some use church activities as an excuse for time spent away from home. Others pretend to go to bed and then sneak out during the night, leaving home around 10 p.m., returning at 4 a.m.

From conversations with YWSS it appeared many experience exploitation, such as when older FSWs offer them a roof over their heads in exchange for a share of their takings. This was one of the few examples of interaction between YWSS and adult FSW. Due to lack of experience, YWSS reported not always being able to negotiate condom use with their clients. YWSS also stated they dislike seeking medical treatment for symptoms of sexually transmitted infections (STIs) out of shyness or lack of knowledge about available services. Importantly, they said YWSS might be unwilling to come for research interviews due to its association with their involvement in sex work.

### Differences across the six sites

Several forms of selling sex were unique to one or few sites. In the larger, more urban locations, party-based sex featured heavily in descriptions of the local YWSS scene. While some parties are simply for personal enjoyment, others have organised sex and drinking competitions. These ‘Chillers Parties’ are usually hosted by younger males, in their parents’ houses at weekends when their parents are out of town. The men pool resources to buy alcohol and gifts for competition winners. Marijuana use is common. Party organisers offer transport to and from the party venue from different parts of town and girls gain entry without payment, while men pay an entrance fee. YWSS go to parties explicitly to meet potential clients but do not usually openly solicit there. Space for sexual encounters is provided and sex takes place in public with party attenders judging different categories, such as “the best kissers” and “the best sex act”. The girls who win the sex competition receive money, make-up kits or other prizes. The ones who win are the ones who have the most sex or exhibit the best ‘moves’. YWSS said that condom use at parties was uncommon.

In the four towns with local universities, it appeared to be common for female students to sell sex without associating it with formal sex work. Key informants described parties and night spots specifically targeting university students. In some cases, male students act as go-betweens for female students and potential clients, many of whom were adult men from outside the university. More established FSW complained students were stealing their business by accepting low or no fees. For example, it was said students exchange sex for a meal, alcohol or to get their hair done. Our discussions with *Sisters* peer educators revealed that students organise large parties, sponsored by a wealthy student or through charging entry fees. In one site, a garage located in an industrial zone far from residential areas becomes a sports bar at night. Parties provide an opportunity for female students to meet “blessers,” a slang term for older men who give young women support in the form of money and materials goods in exchange for companionship, often including sex.

Students also take advantage of functions formally organised by universities. For example, student associations organise welcome parties, Valentine’s Day balls, midyear and term shut down parties, held in local hotels. These run from late evening until dawn, after which students move on to local night spots. During these parties, the female students who sell sex can hire rooms at the venues or sometimes a simple woven mat, *tswanda*, which is placed behind the bar on which to have sex. Clients follow students to parties, and this form of sex work is concentrated during term times.

The rationale given for female student engagement in transactional sex was that parents or guardians pay only tuition fees and not upkeep costs. Female students also described even less formalised types of sexual exchange through having several boyfriends to fulfil different livelihood needs. They labelled these according to material support provided, e.g. “ministry of transport” (gives money for transport during the term), or “ministry of housing” (pays rent). Boyfriends might provide food, clothing, fashion accessories and perfume. Some boyfriends recruit students into additional sex work. For example, one male key informant explained that he might invite a female student to meet him and asks her to bring a group of friends. He will buy drinks for them all, and then later introduces them to his friends for sexual encounters. While the students do not charge a fee, alcohol provides payment and these liaisons can result in longer term “blesser” arrangements.

In two of our study sites there is a significant economy around illegal gold panning. Gold panners earn large amounts of money on a sporadic basis. While gold panners thus pay more than other clients, they are thought to be especially violent towards YWSS. In these locations, both YWSS and older FSW congregate in locations where panners spend time and compete for their business. Finally, in the smaller urban areas, YWSS had migrated in from surrounding rural areas. Many moved to the urban sites in the hope of finding paid work and end up selling sex. These YWSS described themselves as “run-aways” or “school drop outs”. They travel back and forth to their rural homes, sharing rented accommodation in the towns while trying to earn money.

## Discussion

We used “social mapping” as a structured rapid appraisal approach to characterise the YWSS context in six sites over a short period of time, gaining understanding of both geographical and social patterns of how younger women sell sex. We conducted the mapping to collect practical information to inform strategies for ensuring the DREAMS programme reaches YWSS among the broader target population of high risk young women. Our results are also salient for the design of process and impact evaluations, which will need to accommodate differences in how YWSS sell sex, interact, and seek services.

We found many similarities across sites. Most YWSS live outside their city or town’s CBD and travel into town on weekend evenings once their local bars close. They do so to both preserve anonymity and seek out higher paying clients. Those who remain in the high density suburbs charge less money and see themselves as professional FSW. Due to hostility from older sex workers, YWSS avoid bars and other locations with a concentration of FSW, instead relying on attracting clients outside them, taking advantage of looking younger and “new”. This approach, coupled with their tendency to charge less money or accept food or other material goods in lieu of cash, contributed to ongoing conflicts with older FSW. YWSS also fear being identified and hide their work from parents and other community members, making them reluctant to associate closely with one another. Fear of disclosure appears to make them less willing to seek sexual health care, even at targeted services for sex workers such as *Sisters* clinics.

We also identified several important contextual differences across our six study sites. Most notably, the four university towns had unique opportunities for YWSS to transact sex, and university students were the least likely to self-identify as sex workers or perceive their engagement in transactional sex to be high risk. Students did not self-identify as sex workers, and did not congregate in venues typically associated with commercial sex. Nonetheless, their client base extended beyond the student population, potentially overlapping with clients of other sex workers. Perhaps at the other end of the YWSS continuum, migrant young women from rural areas perceived themselves to be school “drop outs” and “runaways” and were more likely to see themselves as professionalised sex workers, seeking out groups of men known to be clients such as illegal gold panners or truck drivers.

Some of our findings reflect existing evidence on YWSS from Zimbabwe and other contexts. For example, YWSS often move from less formal transactional sexual exchange into more highly structured and “professional” sex work over time [9, 10]. They engage in sex work for multiple reasons; some require significant livelihood support, while others seek luxury gifts or disposable income [11–14]. Younger sex workers tend to be fearful of engaging with services out of fear of their involvement becoming known to parents or other community members [15, 16]. Furthermore, hostility between older and younger sex workers exacerbates competition and makes it more difficult for them to collaborate for improving work conditions and reducing vulnerability. For example, in Uganda, younger sex workers also avoided contact with older FSW by working outside bars [17]. Participation of school and university students in transactional sex is widespread and many studies have similarly found students to be unlikely to consider their exchange of sex for financial or material benefits to be sex work *per se* [12, 18, 19].

Our findings also provide new insights on YWSS and their vulnerability to HIV, particularly for the Zimbabwean context. Although CeSHHAR has worked with FSW across the country since 2009, this study is the first time we came across formally organised parties at which YWSS are recruited by same-age or older males specifically for arranging sexual exchange. Both the Chillers parties and the university functions integrate social events for young people with organising transactional relationships with older males. Both events combine on-site, short term sexual encounters with opportunities for YWSS to recruit longer term “blessers” or regular clients. In general, the way in which YWSS sell sex is poorly understood and often excluded from the literature on FSW due to the considerable ethical considerations in including YWSS under age 18 in research [20, 21]. Increasingly, however, there have been calls to move beyond simplistic portrayals of all underage YWSS as “trafficking victims” in order to gather data that are required for effective methods of reaching this vulnerable population, including offering services that are likely to be acceptable and taken up by YWSS, rather than driving them further underground and into higher risk situations [10, 21, 22].

There are thus several implications of our “social mapping” exercise for the DREAMS programme and its evaluation. A comprehensive understanding of the local context in which YWSS work, live, and seek opportunities to improve their lives will help guide suitable messages and interventions. First, even where there are common trends in how YWSS live and work, they remain largely outside existing FSW social networks and do not engage with targeted services. Linking them to DREAMS’ health and social interventions will require intensive measures and cannot rely on leveraging adult FSW peer educators and their familiarity with the community. Second, there are clearly different typologies of YWSS, for example “street based” women who nonetheless cluster in known sex work “hot spots”, female university students who transact sex during term times at social events, and migrant rural AGYW who target concentrations of men with disposable income who are themselves difficult to identify, such as illicit gold panners. These groups of YWSS do not associate with each other, nor would them all self-identify as YWSS to the same degree. Approaches used to engage young women aged 18–24 who consider themselves professional sex workers will need to differ from those who consider their exchange of sex for money or goods to be part of a temporary livelihood strategy. Care needs to be taken in considering which components of the DREAMS package are likely to appeal to each group, and how to ensure referrals are taken up. For example, self-described school drop-outs may find educational subsidies or vocational training the most appealing entry point into DREAMS, while YWSS engaged in selling sex on the street may want youth-friendly clinical sexual and reproductive health services in the first instance. Students may be split between those interested in educational support, job opportunities, or “edu-tainment” type HIV awareness raising campaigns. Following initial, positive contact with DREAMS may subsequently lead to higher uptake of a broader constellation of services.

Finally, the evaluation of DREAMS requires that the baseline context of vulnerability be clearly identified in both intervention and comparison sites, to allow for comparison across time and place. Due to the way DREAMS was designed for Zimbabwe, it was difficult to select similar sites for intervention and comparison. For this reason, the two urban areas where the intervention is being implemented have been paired with 4 smaller towns for comparison. While the numbers of YWSS may be broadly similar, the nature of how they sell sex has some significant differences that need to be taken into account.

### Limitations of approach

Social mapping was conducted in six sites in Zimbabwe and does not necessarily depict all of Zimbabwe’s settlements, hence data collected cannot be generalised. The study was conducted among a relatively small number of young women who sell sex who were willing to participate in informal group discussions. They may not represent behaviours of the majority of young women in the age group of interest. The rapid assessment took place over two weekends at the beginning of the year which has further limitations: universities were on holiday and more time would obviously have enabled us to gather more data. Despite this social mapping reaches hidden populations; less is known about the typology of sex work in YWSS. This paper adds a wealth of knowledge to the makeup of sex work in this relatively young age group, who are often under-represented in studies combining older and younger women selling sex.

## Conclusions

Successful intervention and research design rely on in-depth understanding of local contexts and risk environments. Our social mapping of YWSS across six sites in Zimbabwe, provides one example of applying a structured yet informal rapid appraisal to gather information useful to subsequent recruitment of a hard-to-reach population. Our YWSS matrix and (sample) visual map demonstrate the kinds of outputs generated and may provide a starting point for research into YWSS in other contexts.
